# Antioxidant, cytotoxic and DNA protective properties of *Achillea eriophora *DC. and *Achillea biebersteinii *Afan. extracts: A comparative study

**Published:** 2017

**Authors:** Maryam Varasteh-kojourian, Parvaneh Abrishamchi, Maryam M. Matin, Javad Asili, Hamid Ejtehadi, Fatemeh Khosravitabar

**Affiliations:** 1 *Department of Biology, Faculty of Sciences, Ferdowsi University of Mashhad, Mashhad, Iran*; 2 *Cell and Molecular Biotechnology Research Group, Institute of Biotechnology, Ferdowsi University of Mashhad, Mashhad, Iran*; 3 *Department of Pharmacognosy, School of Pharmacy, Mashhad University of Medical Sciences, Mashhad, Iran*

**Keywords:** Phenol, Flavonoids, DPPH, MTT assay, Comet, HFF3 cells

## Abstract

**Objective:**

*Achillea* is a traditional medicinal herb which contains different phenol and flavonoid compounds that are responsible for *Achillea* pharmacological effects. We aimed to determine phenol and flavonoid contents, besides antioxidant activities of different extracts from *Achillea eriophoraa *(*A. eriophora*) DC. and *Achillea biebersteinii *(*A. biebersteinii*) Afan. (endemic species in Iran) and to investigate their effects on human cells.

**Materials and Methods:**

*Achillea* extracts, were prepared by maceration and shaking methods, from different parts (aerial parts, stem, leaves and inflorescence) of two species using methanol and ethanol as solvents. Total phenol and flavonoid contents were measured by spectrophotometry, and antioxidant activities of the extracts were determined by DPPH radical scavenging, BCB and TBARS assays. Cytotoxicity and antioxidant activities of the extracts were investigated in Human Foreskin Fibroblast (HFF3) cells using MTT, comet and H_2_O_2_ assays.

**Results:**

Methanol extracts of *A. biebersteinii* prepared from leaves and inflorescence by maceration method exhibited maximum phenol (1657.58 ± 36.45 mg GAE/100 g DW) and flavonoid (264.00 ± 62.16 mg QUE/100 g DW) contents. Leaf methanol extract showed significantly higher antioxidant activity (0.0276 ± 0.003, 0.16 ± 0.016 and 13.96 ± 0.26 mg/ml for DPPH, BCB and TBARS IC50s, respectively) than those of the other extracts. Leaf extract of *A. biebersteinii* was not cytotoxic even at the highest examined dose (512 µg/ml) and inhibited cell toxicity induced by H_2_O_2_ (98% viability for the cells pretreated with plant extract in the presence of H_2_O_2_). Comet assay also confirmed high DNA protective activity of leaf extracts.

**Conclusion:**

*Achillea* extracts possess remarkable antioxidant activity, and could be good natural alternatives to synthetic antioxidants in pharmaceutical and food industries.

## Introduction

*A* *chillea*, which belongs to the family Compositae (Asteraceae), is a genus with over 100 species all around the world. Although these medicinal perennial rhizomatous plants are native to Europe and Western Asia, they are also found in Australia, New Zealand and North America (Chevallier, 2000 ▶). The two studied species *Achillea eriophora* (*A. eriophora*) DC. And *Achillea biebersteinii *(*A. biebersteinii*) Afan. are endemic plants in Iran. 

*Achillea*, known as “Bumadaran” in Persian, is one of the most widely used medicinal plants in Iran. It is used as hypoglycemic, nerve tonic, anti-hemorrhoid, anti-diarrhea, antacid, carminative, appetizer, anthelmintic and anti-bacterial remedies (Amiri and Joharchi, 2013 ▶; Ghorbani, 2005 ▶; Pirbalouti and Golparvar, 2007 ▶; Zargari, 1993 ▶). These pharmacological properties have been mainly attributed to the phenolic and polyphenolic compounds, which are well known as antioxidant agents (Evans, 2009 ▶; Harborne and Williams, 2000 ▶; Weber et al., 2006 ▶). Clinical evidences have revealed that antioxidants are effective in the treatment of various diseases, including atherosclerosis, arthritis, ischemia and reperfusion injury of many tissues, central nervous system injury, gastritis, and cancer, and are beneficial to the wound healing process (Cook and Samman, 1996 ▶; Kumpulainen and Salonen, 1999 ▶). These activities are in accordance with reported pharmaceutical properties for *Achillea*. 

Antioxidant activity of different species of *Achillea* including *Achillea millefolium* L. (Candan et al., 2003 ▶), *Achillea ligustica* All. (Conforti et al., 2005 ▶), *Achillea wilhelmsii* (Ozgen et al. 2004 ▶) and *A. biebersteinii *(Tawaha et al., 2007 ▶) have been previously investigated. However, the protective effects of *A. eriophorea* and *A. biebersteinii* against oxidative stress in HFF3 cells have not yet been reported. In this study, two extraction methods and solvents were used to obtain phenol and flavonoid-enriched extracts from different parts of *A. eriophorea* and *A. biebersteinii*. The antioxidant activity of the methanol extracts prepared by maceration from different parts of the plants, was evaluated using different assays. To the extent of our knowledge, this is the first report about the antioxidant activity of leaf methanol extracts of *A. eriophorea *and *A. biebersteinii* in human fibroblast cells.

## Materials and Methods

**Chemicals**

Folin-Ciocaltue, sodium carbonate, methanol, ethanol, gallic acid, aluminum chloride, 1,1-diphenyl-2-picrylhydrazyl (DPPH), beta-carotene, linoleic acid, tween40, chloroform, butylated hydroxyl toluene (BHT), 2,2’-azobis-(2-amidinopropane) dihydrochloride (ABAP), acetic acid, tiubarbituric acid, sodium dodecyl sulfate (SDS), butanol, 3-(4,5-dimethylthiazol-2-yl)-2,5-diphenyl-tetrazolium bromide (MTT), H_2_O_2_, ethylenediaminetetraacetic acid (EDTA), dimethyl sulfoxide (DMSO) and all solvents were purchased from Merck (Germany). Ascorbic acid and potassium acetate were purchased from Sigma–Aldrich (St. Louis, MO, USA). Dulbecco's Modified Eagle's Medium (DMEM) and fetal bovine serum (FBS) were obtained from Gibco Life Technologies (Grand Island, NY, USA).

**Sample collection and extract preparation**

Plants were collected at the flowering stage in May 2011 from pastures of Khorassan Razavi (N 36.291886, E 58.583121; 1618 m above sea level) and South Khorasan provinces (N 36.34020, E 40.710158; 1591 m above sea level), Iran. The voucher specimens (No. 30348 and 22004 for *A. eriophora* and *A. biebersteinii*, respectively) were deposited in Herbarium of Ferdowsi University, Mashhad, Iran.

Grinded dry materials from different parts of plants (stem, leaves, aerial parts and inflorescence), were extracted with methanol and ethanol (1:20 w/v) using maceration and shaking. Extracts were filtered through the regular filter paper and evaporated under vacuum.

**Total phenol and flavonoid assay**

The total phenolic content of all extracts was determined spectrophotometrically according to Folin-Ciocalteu method (Pattanayak et al., 2012), and total flavonoid content was determined using aluminum chloride colorimetric method (Chang et al., 2002 ▶). The results were expressed in terms of gallic acid equivalent (GAE mg/g of dry weight) for phenolic content and quercetin equivalent (QE mg/g of dry weight) for flavonoid content, which are the two common reference compounds.

**Antioxidant assays**

**DPPH radical scavenging microplate assay**

The antioxidant properties of methanol extracts (0.5-7 mg/ml) or gallic acid as a standard, were investigated by reducing the stable DPPH radical (Yang et al., 2011 ▶). The absorbance was then measured at 492 nm using an ELISA reader (Awareness Technology, USA). The antioxidant index (AI %) was calculated as [(1 – *A*_1_ – *A*_2_/ *A*_0_) × 100]. Where *A*_0_ is the absorbance of the control reaction (without sample), *A*_1_ is the absorbance of sample/gallic acid, and *A*_2_ is the absorbance of sample without DPPH. Analyses were run in triplicate, and IC_50_ values (presenting the concentration with 50% antioxidant index) were calculated.

**Beta-carotene bleaching microplate assay (BCB)**

A modified method of Dapkevicius et al. (1998) ▶ was used to test bleaching ability of the extracts against beta-carotene (Dapkevicius et al., 1998 ▶). Briefly, 1 mg of beta-carotene was dissolved in chloroform (5 ml) and then, linoleic acid (25 µl) and Tween 40 (200 mg) were added to 1 ml of this mixture. After removing chloroform using a rotary evaporator at 40ºC, the remaining where solved in oxygenated distilled water (50 ml) and vigorously shacked. An aliquot of 250 µl of the above-prepared beta carotene–linoleic acid emulsion was applied to each well of a 96-well plate. Next, 30 µl of different concentrations of the extracts (0.5-7 mg/ml) or BHT as a standard (1-100 µg/ml) were added to each well in triplicate. An equal amount of the extracts or BHT was used as blank. The microplates were incubated at 55ºC and their optical densities were determined at 492 nm using an ELISA reader. Reading the absorbance of all samples was carried out at the start (t=0) and after 105 min of incubation. The antioxidant activity coefficient (AAC) was estimated according to the following formula:

ACC = [(*A*_T_*105 *-* A*_B_*105*) / (*A*_B_*0*–*A*_B_*105*)]

Where *A*_T_*105* and *A*_B_*105* are absorbance of sample and blank after 105 min, respectively. *A*_B_*0* is the absorbance of blank at t=0.

**TBARS assay**

A modified TBARS assay (Bazzaz et al., 2011 ▶), using egg yolk homogenates as lipid rich media, was applied to measure the antioxidant capacity of the extracts. Briefly, 500 µl of yolk homogenate (10% w/v in distilled water) and 100 µl of sample solution (5-50 mg/ml concentrations of the extracts or standard), were added to a test tube and made up to 1 ml with distilled water, followed by addition of 50 µl of ABAP aqueous solution (0.07 M; for induction of lipid peroxidation), 1.5 ml of 20% acetic acid (pH 3.5) and 1.5 ml of 0.8% (w/v) thiobarbituric acid in 1.1% (w/v) SDS. The mixture was vortexed, and heated at 95ºC for 60 min. After cooling, 5 ml butan-1-ol was added and extensively vortexed and centrifuged at 2500 g for 10 min. The absorbance of the upper organic layer was measured at 532 nm using a spectrophotometer. Butanol and BHT were used as the blank and positive control, respectively. All testes were carried out in triplicate. Values were expressed as antioxidant index (AI%) according to the following formula:

AI% = (1 –* t */ *c*) × 100

Where *t* and *c* are the absorbance of the test sample and the fully oxidized control, respectively.

**Cell culture**

**Cytotoxicity assay**

HFF3 (Human Foreskin Fibroblast) cells (a generous gift from Royan Institute, Tehran, Iran) were seeded at 8000 cells/well in 96-well plates in DMEM supplemented with 10% FBS. After 24 h of incubation in a humidified 5% CO_2_/air environment at 37ºC, when cells became 70-80% confluent, they were treated with different concentrations of the extracts (1-512 µg/ml) diluted in DMEM containing 1% FBS. Following 24, 48 and 72 h of incubation with the extracts, culture media were aspirated and replaced with DMEM containing 1% FBS and 20 µl of MTT solution (0.5 mg/ml). After 4 h of incubation, media were removed and purple colored crystals were dissolved in DMSO. Absorbance of each well was measured at 450 nm using an ELISA reader. 

**Antioxidant activity of plant extracts on HFF3 cells**

The hydrogen peroxide (H_2_O_2_) assay was modified to assess the protective effects of the extracts in HFF3 cells against the oxidative damage induced by H_2_O_2 _(Kumar and Gupta, 2011 ▶). Fibroblast cells were seeded in 96-well plates at 8000 cells/well in DMEM containing 10% FBS, and grown at 37ºC to near confluence. Cells were serum-deprived and pretreated with leaf extracts 1 µg/ml for 2 h. Pretreated cells were then exposed to different concentrations of H_2_O_2_ (10, 100, 250, 500 and 1000 mM), for 24 h. The degree of protection of fibroblast cells by extracts against H_2_O_2_ damage was then quantified by MTT assay.

**Comet assay**

Single cell gel electrophoresis was performed (Rassouli et al., 2011 ▶) on HFF3 cells treated with H_2_O_2_ and leaf extracts. HFF3 cells were grown in DMEM containing 10% FBS in 6-well plates, for 24 h. The attached cells were pretreated for 2 h with 5 ml DMEM (1% FBS) containing leaf extracts 1 µg/ml of both species. After this pretreatment, H_2_O_2_ (500 mM) was added and cells were incubated for 24 h. Cells were then washed with 5 ml cold PBS, and detached by trypsin/EDTA solution for further analysis. Each data point represents the average DNA in tail of at least 150 measurements (comets). Comets were analyzed with Tri Teck Comet Score version 1.5.

**Statistical analysis**

Phenol and flavonoid contents were analyzed using univariate ANOVA, and Duncan as multiple range mean comparing test. One-way ANOVA was used to analyze the IC_50_ values of antioxidant testes, means of H_2_O_2_ and comet assay results. Also, Duncan was performed as *post-hoc* analysis. Results were expressed as mean ± S.D. All analyses were performed using STATISTICA (Statsoft, 2011) software.

## Results

**Total phenol and flavonoid contents**

Total phenol and flavonoids content in the *Achillea* species ranged from 149 to 1657 mg gallic acid equivalent (GAE) /100 g dry weight, and 59-264 mg quercetin equivalent (QUE) /100 g dry weight, respectively (Table 1). The highest total phenol content was shown by inflorescence extract followed by leaf extract of *A. biebersteinii*. Also, the highest total flavonoid content was recorded for leaves extract of both species. Methanol extracts prepared by maceration method, possessed higher phenol and flavonoid content as compared to those prepared with ethanol and shaking methods. 

**Table 1:** Effect of extraction methods (maceration and shaking) and solvent type on total phenol and total flavonoid content in different parts of *Achilea eriophora* and *Achilea biebersteinii* from Iran

**Plant organ**	**Solvent**	**Total phenolmg GAE/100 g dry plant tissue**	**Total flavonoidmg QUE/100 g dry plant tissue**
**Stem (** ***A. eriophora*** **)**	methanol	316.79 ± 43.32	92.13±4.02
		278.02 ± 110.33	88.12±8.48
	ethanol	229.93 ± 9.02	70.04±2.75
		225.37 ± 37.59	59.25±4.30
**Leaves**	methanol	1050.82 ± 184.20	244.06±1.27
		890.17 ± 75.27	232.08±8.23
	ethanol	622.76 ± 138.90 740.92 ± 84.48	226.42±1.71 196.58±6.71
**Aerial parts**	methanol	667.74 ± 73.30	216.45±2.15
		581.09 ± 98.45	201.69±44.36
	ethanol	327.15 ± 46.80	93.81±2.89
		365.29 ± 142.42	81.83±12.99
**Inflorescence**	methanol	812.23 ± 64.39	216.56±1.67
		807.46 ± 105.01	190.13±16.35
	ethanol	570.72 ± 51.53	156.97±4.88
		566.16 ± 55.75	133.89±10.76
**Stem (** ***A.biebersteinii*** **)**	methanol	293.36 ± 77.24	77.79±4.40
		284.45 ± 51.04	74.00±4.09
	ethanol	149.91 ± 16.39	74.19±7.17
		164.84 ± 12.84	71.01±1.54
**Leaves**	methanol	1168.98 ± 146.10	249.99±12.01$
		1156.34 ± 76.92	264.00±62.16$
	ethanol	694.48 ± 56.81	236.36±3.25
		719.15 ± 26.30	207.82±5.08
**Aerial parts**	methanol	848.30 ± 121.90	203.86±6.63
		821.14 ± 51.98	210.21±21.79
	ethanol	454.02 ± 57.88	214.91±4.00
		389.13 ± 81.48	188.15±1.93
**Inflorescence**	methanol	1657.58 ± 36.45*	226.09±4.83
		1441.79 ± 215.27	197.49±28.53
	ethanol	1347.47 ± 61.37	184.99±8.13
		1228.68 ± 50.85	169.25±6.73

In each row, presented data belong to maceration and shaking methods, respectively.

*, $ p<0.05. Data are means ± SD of five replicates.

**Evaluation of antioxidant activities by different methods**

Since among all the extracts, leaf and inflorescence methanol extract showed the highest phenol and flavonoid contents, maceration extraction and methanol solvents were selected for evaluation of antioxidant activities. The antioxidant capacity of *Achillea* extracts, as presented in Table 2, were determined by the following three methods: DPPH, BCB and TBARS assays. According the three different assays, leaf extract of *A. biebersteinii*, showed the highest antioxidant activity in three selected assays. IC_50_ values for leaf extract of *A. biebersteinii* were 0.27, 0.16 and 13.96 mg/ml, in DPPH, BCB and TBARS tests, respectively. Inflorescence extract of *A. eriophora* showed the lowest DPPH radical scavenging activity. Also, at concentrations below 100 mg/ml of inflorescence extracts in both species, TBARS AI did not reach 50%.

**Effects of** ***Achillea*** **leaf extracts on viability of HFF3 cells**

**Figure 1:**
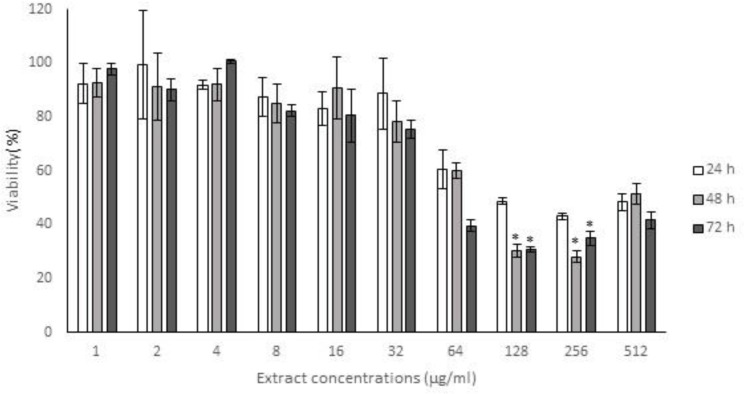
The cytotoxicity of the methanol extract of the leaves of *Achilea eriophora* on the proliferation of human foreskin fibroblast cells (HFF3). The cells were treated with various concentrations (1-512 µg/ml) of the extract in a culture medium for 24, 48 and 72 h. Cells viability was determined and compared with the control (untreated cells) by the MTT assay. Each value represents the mean ± SD (n=3). *p<0.05 Starred values, are significantly different from other means

**Figure 2:**
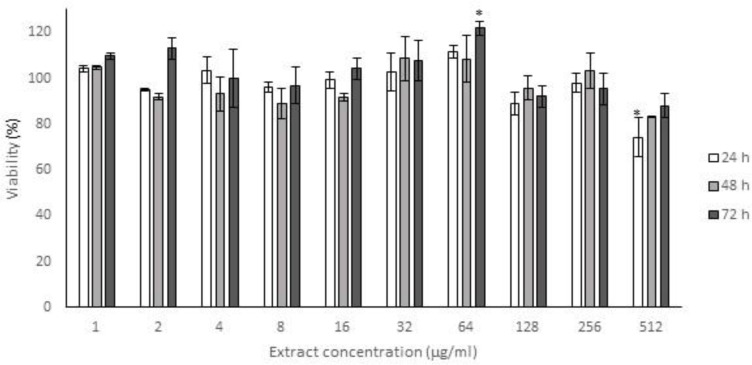
The cytotoxicity of the methanol extract of the leaves of *Achilea biebersteinii* on the proliferation of human foreskin fibroblast cells (HFF3). The cells were treated with various concentrations (1-512 µg/ml) of the extract in a culture medium for 24, 48 and 72 h. Cells viability was determined and compared with the control (untreated cells) by the MTT assay. Each value represents the mean ± SD (n=3). *p<0.05 Starred values, are significantly different from other means

Antioxidant capacity and cytotoxic effect of methanol extracts of *Achillea* leaves were examined *in vitro*. Viability of HFF3 cells treated with different concentrations of *A. eriophora* extracts, reduced in a dose-dependent manner, but the highest examined dose (512 µg/ml) of *A. biebersteinii* extract showed no toxic effects. IC_50_ values obtained for *A. eriophora* extract were 120, 85 and 55 mg/ml after 24, 48 and 72 h treatments, respectively (Figures 1 and 2).

**Table 2:** Antioxidant activities of the Achillea extracts

**Plant Species **	**Plant organ **	**IC50 (mg/ml)**		
		**DPPH**	**BCB**	**TBARS**
***Achillea eriophora***	Leaves	0.703±0.023 b	1.46±0.03 a	23.83±0.5 a
	Inflorescence	0.91±0.001 a	1.78±0.441 a	> 100
***Achillea biebersteini***	Leaves	0.276±0.003 d	0.16±0.016 b	13.96±0.26 b
	Inflorescence	0.33±0.006 c	1.63±0.176 a	> 100
**Ascorbate**		0.016±0.0003 e	-	-
**BHT**		-	0.00015±0.00002 b	4.93±0.74 c

Data are mean ± SD of three replicates.

a-e , means in each column following different letters are significantly different (p<0.05) as determined by Duncan’s multiple rang test.

**Antioxidant activity of leaf extracts on HFF3 cells**

**Figure 3:**
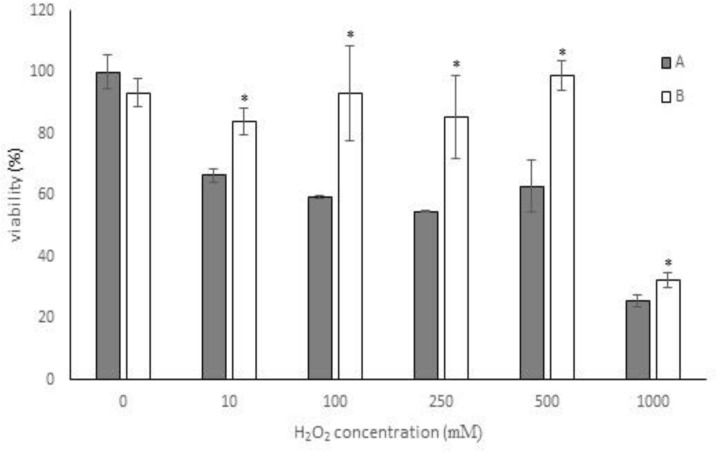
The effect of methanol extract on H_2_O_2_ cytotoxicity in human foreskin fibroblast cells (HFF3). Cytotoxic effect of H_2_O_2_ at 0-1000 mM concentrations (A) were compared to cells pre-treated with 1 µg/ml *A. biebersteinii* leaves extract (B). Cells viability (%) was determined and compared with the control (untreated cells) using MTT assay. Each value represents the mean ± SD of three replications. *p<0.05 compared with the corresponding concentration of H_2_O_2_ without plant extract pre-treatment

**Figure 4:**
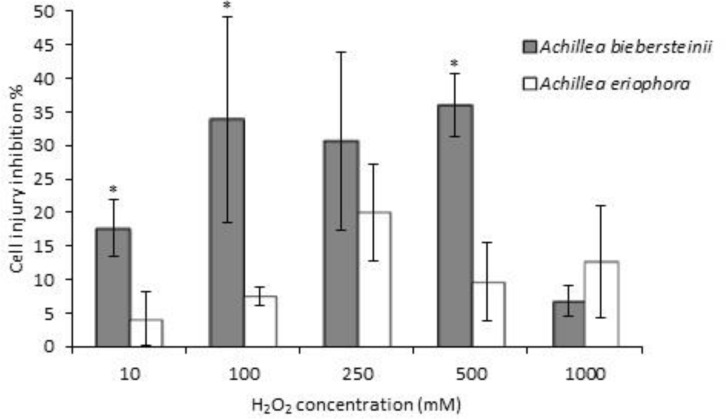
*Achillea* extracts inhibited HFF3 cells oxidative injury. HFF3 cells injury induced by H_2_O_2_ were inhibited by pretreatment with *Achillea* extracts (1 µg/ml). The percentage of cell injury inhibition was calculated on the basis of viability using MTT assay. Data are mean ± SD of three replicates. *p<0.05 compared with corresponding concentration of H_2_O_2_ with different plant extract pre-treatment

*Achillea* leaf extracts were selected due to their high phenol content and antioxidant activity, and their possible protective effects against H_2_O_2_-induced damages were investigated *in vitro*. The protective effects of the extracts on HFF3 cells were first confirmed by MTT assay. Reduced viability was recorded at different concentrations of H_2_O_2_ (54.73% at 250 mM H_2_O_2_, Figure 3), but pre-treatment with 1 µg/ml of the *Achillea* leaf extracts, could significantly (p<0.05) inhibit oxidative damage (Figure 3). Also results showed that pre-treatment with *A. biebersteinii* leaves extract were more effective than pre-treatment with *A. eriophorea* to inhibit cell injuries caused by H_2_O_2_ treatment (Figure 4). The oxidative damage to the DNA of HFF3 cells was examined using comet assay in H_2_O_2_-treated cells.

**Figure 5:**
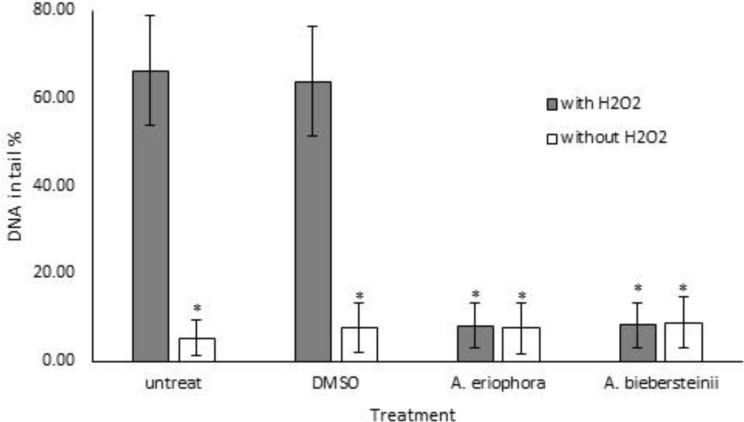
Effect of methanol extracts from the leaves of two *Achillea* species (1 µg/ml) on DNA damage induced by H_2_O_2_ (500 mM) in human foreskin fibroblast cells (HFF3) assessed by Comet assay. *A. eriophora* and *A. biebersteinii* groups were pre-treated with different extracts (1 µg/ml), DMSO group was pretreated with DMSO and untreated group was the null control without any pretreatment. DNA in tail in each cell was calculated by comet score. The data represent mean±SD of 150 cells. *p<0.05 Starred values compared with means in untreated and DMSO groups which are treated with H_2_O_2_

**Figure 6:**
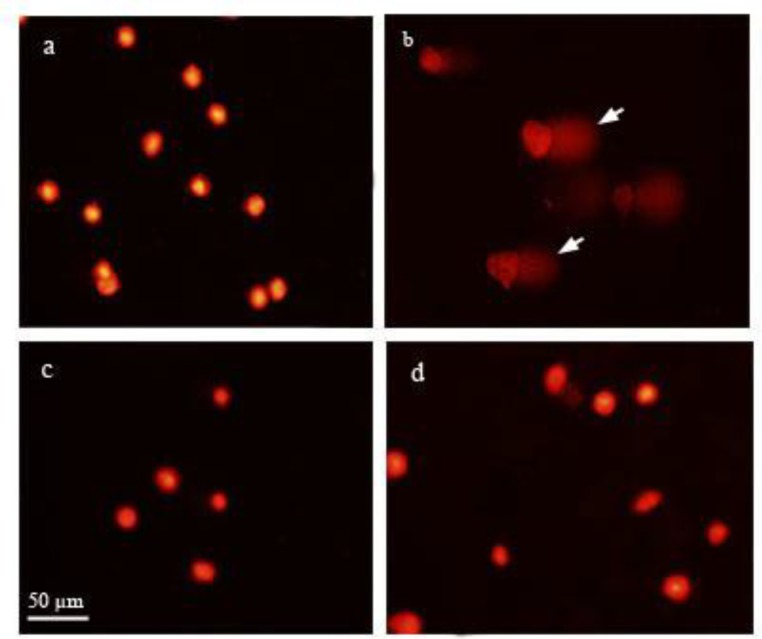
Comet tails of human foreskin fibroblast cells (HFF3) treated with H_2_O_2_ and *Achillea* extracts. Pre-treatment with the methanol extract of *Achillea* leaves (1 µg/ml) significantly (p<0.05) reduced comet tail of HFF3 cells induced by H_2_O_2_ (500 mM) treatment. Untreated cells (a), H_2_O_2_ treated cells (b), H_2_O_2_-incubated cells pretreated with *Achillea eriophora* (c), and *A. biebersteinii* extracts (d). Arrows indicate comet tails which contain damaged DNA

Comet results also confirmed protective effects of leaf extracts (1 µg/ml) against H_2_O_2_-induced oxidative damage. As shown in Figure 5 and 6, DNA in tail was reduced in cells pretreated with *Achillea* extracts after H_2_O_2_ treatment (8.9% and 7.6% for *A. biebersteinii* and *A. eriophora*, respectively), as compared to H_2_O_2_-treated cells in which the DNA in tail was about 66%.

## Discussion

There are different reports on the total phenolic contents of the leaf extract of *A. biebersteinii*. For example, Tawaha et al. reported that this value varied between 1600 and 2300 mg of GAE/100 g (Tawaha et al., 2007 ▶), while in the study of Sokmen and Özbek, it was calculated as 51 ± 0.6 mg/g (Sokmen and Özbek, 2006 ▶). These differences may be mainly due to diversities of the analytical methods, extraction methods, variety, developmental phase, and geographic origin of the plants. Similar to our results (Table 1), there is a study that reported the total phenol contents of *A. eriophora* to be were higher in leaves than inflorescence (Dokhani et al., 2005 ▶). 

As indicated in the results, leaf extracts of *A. biebersteinii* with the highest antioxidant activity (as measured by three different methods), showed the highest phenol and flavonoid contents as well. It seems that higher antioxidant activity of *A. biebersteinii* extracts, as compared to *A. eriophora* extracts, could be attributed to their higher phenolic and flavonoid contents. 

This study demonstrated that the cytotoxicity induced via oxidative stress (H_2_O_2_-induced stress) could be suppressed by pre-treatment with *Achillea* leaf extracts (1μg/ml). Protective effects of plant extracts against oxidative cell damage, have been previously reported by various researchers (Gião et al., 2010 ▶; Konyalioglu and Karamenderes, 2005 ▶; Yoo et al., 2008 ▶). Adetutu et al. (2011) ▶ and Annan and Houghton (2008) ▶ reported that fibroblast cells were protected against H_2_O_2_-induced oxidative damage using *Bridelia ferruginea* Benth*. *and *Gossypium arboretum *L., and *Ficus asperifolia* Miq. Extracts (Adetutu et al., 2011 ▶; Annan and Houghton, 2008 ▶). Adetutu et al. reached 82% protection, when the concentration of plant extract was 250 µg/ml (in the presence of 180 µM H_2_O_2 _as an oxidant), and Annan and Houghton reported 58% protection against oxidative damage at 50 µg/ml of *F. asperifolia* extract (in the presence of 100 µM H_2_O_2_); However, we observed 35.94% protection at a low concentration (1 µg/ml) of *A. biebersteinii* leaf extract (in the presence of 100-500 mM H_2_O_2_).

 Anti-superoxide properties of flower infusion of *A. biebersteinii* in erythrocytes and leucocytes were reported. Cellular damages induced by 10 mM H_2_O_2_, were blocked after treatment with 500 µl of the infusion. Phenol and flavonoid compounds of infusion were responsible for the positive effects on enzymatic antioxidant system (Konyalioglu and Karamenderes, 2005 ▶). Behravan et al. (2011) ▶ reported a significant inhibitory effect of aqueous extract of *Portulaca oleracea* L. at 1 and 2.5 mg/ml on H_2_O_2_-induced DNA damage in human lymphocyte (percentage tail DNA 2.35% ± 0.16 and 1.29% ± 0.12, respectively). Pretreatment with *Mentha arvensis* L. (25 μg/ml), exhibited a significant protective effect (12.43% of tail DNA% compared to the control) in lymphocytes (Lin et al., 2013 ▶). As *Achillea* species are effective antioxidants, according to the result of antioxidant assays (DPPH, BCB, TBARS), our results suggest that it is likely that the inhibitory effect of *Achillea* on H_2_O_2_-induced DNA damage could be the result of interactions of different antioxidant compounds in the extracts, as reported for *P. oleracea* and *M. arvensis* (Behravan et al., 2011 ▶; Lin et al., 2013 ▶).

Although this study revealed remarkable antioxidant properties of *A. biebersteinii* and *A. eriophora* extracts, further studies are required to determine the chemical composition of the extracts and antioxidant activities of phenolic acids and flavonoids, as their main constituents. Other obvious important topics of research will be to determine the most effective compounds and their mode of action in cell-based assays.

*A. eriophora* and *A. biebersteini*, with remarkable antioxidant activities, especially on human fibroblast cell culture, could be considered as good sources of natural antioxidants and healthy replacements for the corresponding synthetic ones such as industrial food preservatives. Their high phenol and flavonoid contents might be responsible for the traditional usage of these medicinal plants; however, we recommend more detailed studies to determine their exact chemical composition and mechanism of action.
